# Exploring the Sexuality of African American Older Women

**DOI:** 10.9734/BJMMR/2014/5491

**Published:** 2013-11-08

**Authors:** Luciana Laganá, Theresa White, Daniel E. Bruzzone, Cristine E. Bruzzone

**Affiliations:** 1 Department of Psychology, California State University Northridge, 18111 Nordhoff Street, Northridge, CA 91330-8255, USA; 2 Department of Pan African Studies, California State University Northridge, 18111 Nordhoff Street, Northridge, CA 91330-8255, USA; 3 Antioch University Los Angeles, 400 Corporate Pointe, Culver City, CA 90230, USA

**Keywords:** African American, older women, sexuality, sexual desire, methodological limitations

## Abstract

**Aims:**

To identify sexually-related themes of the sexuality of older African American women.

**Study Design:**

Mixed method.

**Place and Duration of Study:**

Department of Psychology, California State University Northridge, between July 2009 and June 2011.

**Methodology:**

We included 13 African American older women (57 to 82 years of age), 11 of whom self-identified as heterosexual, one as bisexual, and one as lesbian. We used a semi-structured interview protocol through which we explored some aspects of the respondents’ sexuality (assessed at a superficial level, to be as tactful as possible). Moreover, we collected information on demographics and self-rated physical health. Two co-authors served as coders, and used content analysis to identify the most salient sexuality themes.

**Results:**

Emerging themes were (in order from most to least endorsed): having sexual desire (often unfulfilled); engaging in less sexual activity in older age; experiencing changes in one’s sexual life as a function of absence of a spouse; and exercising control over how one’s sexual life is conducted. Motivated by the paucity of our sexuality data, we have also provided suggestions to scholars interested in conducting more in-depth further research on this topic with older African American women.

**Conclusion:**

Our findings indicate that the common notion that older women are asexual is a myth, while lack of a suitable sexual partner is a problem reported by many African American older women who would otherwise enjoy sexual interaction.

## 1. INTRODUCTION

There are many erroneous assumptions regarding the sexuality of older adults in general. People often mistakenly believe that interest in sexual activity decreases with age [1]. Unfortunately, the classic misconception that older individuals should not engage in sex [2] sets a negative tone regarding older women’s sexuality. In addition, the needs of family members can significantly drain emotional energy in older couples [3], leaving little or no time for sexual satisfaction. A limited amount of research is currently available on this issue, especially concerning ethnic minority older women. For a variety of reasons detailed below, we chose African American older women as the units of analysis of the study. Concerning the structure of this article, we have 1) provided a succinct literature review on the topic of sexual health among African American older women, 2) described our mixed method study on this topic, and 3) motivated by the lack (or extreme shortness) of the sexuality responses provided by our research participants, described our methodological challenges and offered potential solutions for interested researchers. Hopefully, scholars intending to shed more light on this challenging research area will be in the position to choose from a variety of effective methodological strategies that would allow them to collect richer, more in-depth sexuality data on older African American women.

Concerning sex research on aging populations, there are very few large studies on sexuality in relation to critical factors such as advanced age and health status. Among them, we refer the interested reader to comprehensive research conducted by Tessler Lindau et al. [4] on the sexual a) activity, b) behaviors, and c) problems of a national probability sample comprised of 3,005 adults, age 57 to 85, in which age and health status were related to sexuality issues. This study is particularly relevant to our discussion because African Americans were one of the very few oversampled groups. Briefly, as reported by Tessler Lindau and colleagues, sexual problems most commonly experienced by older women were low sexual desire (43%), vaginal lubrication problems (39%), and difficulties reaching orgasm (34%). Women with poor health were rarely sexually active and, if they did engage in sexual activities, often reported problems with sex. Indeed, without good *physical* health, it is challenging to achieve good *sexual* health in older age, given that both advanced age and poor physical health are risk factors for many sexual problems [5–7]. The latter could also be reflective of specific physical illnesses including urogenital tract conditions, cancer, or major infections [8–9]. Unfortunately, if sex problems are untreated, they might cause other significant problems such as social withdrawal or depression [10].

### 1.1 Issues Related to the Sexuality of Older African American Women

The importance of understanding the sexuality of older African American women in particular (who are the target population of our study) is evident when we consider that, by the year 2030, African Americans will represent 10% of the United States (U.S.) population over the age of 65, and the majority of this population will be women [11]. Unfortunately, this demographic discrepancy between men and women has current (and future) negative repercussions on older heterosexual women’s partner availability and access to a sexual companion, as discussed later. In the present study, we intended to explore the sexuality of African American older women as tactfully as possible. Of particular interest was the identification of specific sexual themes that are most significant in the sex lives of the research participants. The review of prior studies highlighted herein provides a glimpse into some of the many possible themes that could emerge when asking our research participants to express their thoughts and feelings regarding their sexuality.

Ideally, to gain a more in-depth understanding of the sexual lives of older African American women, scholars should consider the unique, larger structural and institutional frameworks on which these women’s sexuality is based, specifically the social, political, and economic contexts of African American life. However, given space limitations, an extensive analysis of the socio-political aspects of African American women’s sexuality is beyond the focus of the present discussion. Readers interested in issues such as how the institution of slavery has impacted African and African American women’s sexuality are referred to insightful work on this topic conducted by scholars such as Wyatt [12–14], who wrote about Sarah Baartmann, better known as Venus Hottentot, considered by many to be the original icon of African American women’s sexuality.

Many scholars have pointed out that there are several difficulties inherent in conducting in-depth research on such an intimate subject in populations of all ages. Among them, Hines [15] argued that many African American women, to best cope with pressure involving their sexuality, engage in the dynamic of dissemblance. This phenomenon consists of creating an appearance of being open to disclosing intimate issues about themselves as well as their feelings; yet, in actuality, they remain enigmatic (as further confirmed in the current study). Additionally, Hines added that African American women were forced to seek protection of their self-image and sexuality due to an unsupportive, antagonistic, and patriarchal White middle class American society. Moreover, as a form of resistance to the negative stigmas and caricatures about their morality, African Americans adopted what Higginbotham [16] coined as the “politics of respectability.” Claiming respectability, through manners and morality, furnished an avenue for African Americans to assert their will and agency to redefine themselves outside prevailing racist discourse. Unfortunately, as Clarke [17] highlighted, the overt acceptance and internalization of strict Puritanical ideals regarding sex (i.e., that sex is to be for the purposes of procreation between married heterosexual couples) among African American women can inhibit their ability to express non-traditional or non-heterosexual desires.

Regrettably, through propagandized images, African American women have been routinely assailed with a unique brand of stereotyping. Not only is she typically labeled as an inadequate, unfeminine woman; but, even African American intellectuals have deemed her as antithetical to the African woman and harmful to African Americans [13]. Importantly, White added, African American women are often portrayed as emasculating of African American men, overbearing to their families, as well as sexually and emotionally abandoned due to their general unattractiveness. Also, they have typically been held responsible for the dysfunctionality of African American families. Through generations of this ideology perpetuated through socialization, media, and social policy, many African American women may have come to internalize these manipulative debasements. In this regard, of particular relevance is the fact that older African American women who came of age during a period of racial segregation are possibly still seeing themselves in the very negative images painted of them at that time. According to White [18], these women seem to have internalized the condemnation and dismissal of their own sexuality, thereby typically forfeiting their sexual empowerment and liberation. In 2004, Mitchem [19], reflecting on patterns of socialization, contended that African American women are judged for their sexualities, when sexuality, like prayer, is personal.

Additionally, issues of financial and economic stability are a significant concern for older African American women. Calasanti [20] stated that aging is experienced as an oppressive life stage by most old people who, due to a lack of income as well as decreased abilities and opportunities, must often rely on support from family and/or governmental resources. This insidious form of ageism (i.e., a prejudice held by people of *all* ages against people who are old) results in, and adds to, cultural negativism about what older adults should do about sex. Therefore, omission of sex and romance among older populations is likely to be, at least in part, a reflection of ageism. Ageism is fraught with all the hazards of the more familiar prejudices of racism and sexism. Butler and Lewis [21] observed that ageism in relation to sex is the ultimate form of desexualization, as sexuality is generally thought of as something that only the young possess. This concept is perpetuated in many forms of mass communication in the U.S. An example of this message deals with the concept of intimacy and how it can be used interchangeably in the minds of many older people as an acceptable substitute for sex. Indeed, intimacy is a major aspect of sexuality and does not always require sexual intercourse; ironically, the latter is typically mistaken for intimacy. Older women often consider the intimacy of close friendship and family ties as more important and fulfilling than having sex. This point was made clear in an American Association of Retired Persons (AARP) study, wherein half of a sample of women age 45+ agreed that sexual activity is a pleasurable, although not necessary, part of a good relationship [22]. However, in a likely effort to counteract and cope with the aforementioned negative pressures and circumstances, African American women have traditionally used African American families and community institutions as places where they could develop agency, as well as a sense of sexual freedom and self-empowerment.

### 1.2 The Present Investigation

The above considerations highlight the need to conduct research targeting the sexuality of older African American women, in an attempt to understand their sexual concerns. In a study on 15 Caucasian women (ages 62 to 79) that inspired the present investigation, Dickson, Hughes, and Walker [23] discussed the dynamics of later-life dating and the search for a long-term mate. Although the men whom these women dated sought intimacy, sex, self-disclosure, and the potential for marriage in their dating partners, women did not always agree, tending instead to describe their dating within the constructs of a) the need for independence and companionship, and b) gender role conflicts in reference to dating. Additionally, increased self-esteem and peer identity were listed by older women as benefits of dating. However, even though they sought companionship, intimacy, and sex, they were very protective of their independent status, both physically and financially, and resisted marriage, even at the risk of losing their partners. Women also felt that their partner’s insistence on remarrying was related to men’s need to be cared for, a situation that many women wanted to avoid.

In our investigation, we intended to conduct research somewhat complementary in scope to that of Dickson et al. [23] by focusing on sexuality in particular, while recruiting a sample of African American older women of a similar size. We used Engel’s [24–25] biopsychosocial model as the conceptual foundation for the present research. This theoretical framework depicts health—including sexual health and well-being—as being related to a multitude of variables. We planned to collect demographic and health status information to provide a picture of our sample in terms of variables such as general health as it related to sexuality. For instance, in the case of marital status, we intended to relate some sex responses to whether a participant was married or single/widowed. No specific a priori hypotheses were made on the outcomes of this research, as very little prior evidence existed in this area to corroborate potential hypotheses. Though we did not make specific hypotheses, we formulated the following two research questions: 1) what are the descriptive statistics on socio-demographic and health variables of our sample? and 2) what are the main themes of the sexuality of the older African American women recruited for this study? Not developing explicit hypotheses in an exploratory, mixed method study with strong qualitative elements of this kind is in line with the methodological requirements of grounded theory [26], as it utilizes content analysis. In addition, we planned on collecting some quantitative data to quantify characteristics of the sample potentially related to sexuality, such as demographic and physical health information.

## 2. MATERIALS AND METHODS

### 2.1 Sample

We gathered a sample of 13 African American older women. Research assistants (RAs) recruited them as volunteers at a variety of community locations, including churches, libraries, and stores. Employing RAs in research on older women has been done in other studies targeting older women’s sexuality (e.g., by Laganá & Maciel [27] in 2010). We used purposive sampling, i.e., our efforts were concentrated on recruiting older African American women within the Los Angeles County’s communities near California State University, Northridge (CSUN). Although RAs worked several hours per week to recruit participants, it took four months (an academic semester) before we were able to locate and interview 13 women who were open to discussing sexual issues. Besides respondents’ willingness to talk about their sexuality, inclusion criteria comprised being at least 55 years of age (in line with many of the aforementioned studies), female, and African American.

### 2.2 Procedures

The CSUN Institutional Review Board approved this study, which was conducted in full compliance with the ethical standards regarding research on human participants. We utilized instruments and procedures aimed at maximizing respondents’ comfort with this research, taking into account Alvidrez and Arean’s [28] recommendations concerning the need for ethical awareness when conducting studies on minority ethnic groups. Most importantly, to this end, we carefully matched RAs to respondents based on both gender and race. RAs were trained by one of the principal investigators of the study in qualitative and quantitative research methods. Participants signed a consent form prior to starting the assessment, which took between 30 and 45 minutes to complete. We asked research participants to select a convenient location where to conduct the interviews; they usually chose places such as senior centers or libraries, as well as their homes. We did not collect identifying information on any participant; each woman was identified only by a random number between 1 and 500, which was put on her research packet once assessment began.

### 2.3 Variables Assessed and Corresponding Instruments

#### Demographics

They were quantified through utilization of a simple list that was created by one of the authors. This list contains 10 items that inquire about respondents’ demographic characteristics such as age, educational level, place of birth and residence, employment status, marital status, and income.

#### Perceived Health Status

An abbreviated version of the Medical Outcomes Study Short Form Health Survey (MOS SF-36), including a self-reported health item and questions assessing respondents’ ability to engage in activities of daily living, was used to quickly assess health status. The entire measure has eight scales, four on mental health and four on physical health, which afford the quantification of several health concepts, including limitations in physical, social and role activities, vitality, and general health perception [29]. Clinical tests of its validity conducted through component analysis have obtained excellent results [30].

#### Sexual Perceptions and Practices

We used a brief, sexuality-focused structured interview protocol created by the principal investigator of the study —as part of an extensive protocol previously used in research by Laganá and Maciel on the sexuality of older Latinas [27]—to explore respondents’ sexual beliefs, self-perceptions of sexual health, and sexual practices. Sample questions are: “Are you sexually active? If not, would you like to be?” and “To whom can you turn if you feel sexually deprived?” In an effort to tactfully elicit sexual information without appearing disrespectful, and to minimize offending respondents, we did not ask questions on specific sexual topics, such as interactional sexual activities or masturbatory practices. As a result, we gathered only somewhat general information on several aspects of the sexuality of our respondents.

### 2.4 Analytic Strategy

Using a mixed-method approach, we calculated descriptive statistics on the socio-demographic variables utilizing SPSS. Health-related answers were used to identify the perceived health status of each participant and were categorized as 1) reported health status and 2) ability to complete activities of daily living independently. Two of the authors of this article transcribed all hand-written protocols (from the RAs who collected the data), typed the content of each interview into Word files and coded the data. Content analyses of the protocols were conducted by these authors, and inter-rater reliability was calculated using Cohen’s Kappa [31]. Each coder was asked to evaluate pertinent data individually by reading and re-reading the protocols several times in order to identify the most endorsed sexuality-related themes. Using content analysis is appropriate for this study, as this has been the method of choice in prior empirical research on sexuality in older age [27] and it conveniently allows for the identification of themes related to sexuality.

## 3. RESULTS

Answers on all themes were typically very short and, at times, consisted of only one or two words; the answers were often intertwined concerning which theme was covered, as many times responses were applicable to two or more themes at once. Regarding the themes (1) having sexual desire and (2) engaging in less sexual activity in older age, overall, the women in this sample responded positively to such questions. Only four of the 13 women reported no sexual desire. One woman emphatically stated “No desire since (my) husband died.” Many respondents indicated that a) their current sexual drive was at a lower level than it had been when they were younger and/or that b) they engaged in less sexual activity due to the aging process or due to health challenges. One participant stated “Of course I do (have sexual desire), but it’s not like I was when I was younger;” another woman declared “Yeah, things (regarding sexual activity) changed because of my health. It doesn’t really bother me.” Most women thought that their sexual desire level was appropriate for their age. One woman reported that her sex drive and sexual activity level had stayed the same throughout her life span (when asked if they had changed with age, she responded “Not really … I have never been very sexual”). No one in this sample verbalized experiencing sexual dysfunction (two women answered “N/A”, and one did not answer this question). Participant number 13, when asked whether she had sexual problems, candidly answered “I have no sexual problems. My only problem is trying to find someone to have sex and to be intimate with.” A respondent who was single, when asked whether she was sexually active, answered “I am not seeing anyone right now, but I would like to be in a relationship.” Seven women reported neither sexual activity nor a desire to engage in interactional sexual activities at this time in their life. Only about a quarter of the sample was sexually active and wanted to continue engaging in sexual activity. A respondent shared that she was pleased with the fact that sexual activity had decreased with advanced age yet its quality was better [“(Sexual activity) has slowed down, but (it is) better as (I have grown) older … (Now it is) more meaningful.”] Conversely, another woman shared her dissatisfaction with the changes in her sexuality as she grew older, stating “Well, the body just doesn’t respond the way it used to.”

Concerning themes (3) experiencing changes in one’s sexual life as a function of absence of a spouse and (4) exercising control over how one’s sexual life is conducted, the majority of the women reported either that they did not have a sex partner or that sexuality questions did not apply to them, as most of them (seven) were widowed. Three of these women perceived that they were not sexually deprived. One woman, when asked if she had any sexual desire, answered “No, not really … I miss my husband, though.” Most of the women without a sexual partner had found ways to be in control of their sex lives, possessing coping skills – covered in theme 4 – to deal with feeling sexually deprived (which was applicable to some unmarried respondents). For instance, answers to “Whom can you turn to if you feel sexually deprived?” included “Prayer” and “(Spending time with) friends.” Some women felt that their sex life was not within their control in that they had sexual desire, but did not have the time, energy, or opportunity to find an intimate companion. To provide an example, a woman answered that she had no one to turn to when she desired sexual gratification [“(I have) no one at the moment because I am not dating, so as for now, no one (can take care of my sexual needs).”] Often, there was a discrepancy between the answers on having sexual desire and those on being sexually active or longing to be sexually active, which was explained by theme 3, as the lack of sexual desire and of sexual interaction was typically attributed to the absence or loss of a partner/spouse.

Although most respondents felt sexual desire, many of these women did not want to engage in any interactional sex with anyone but their husbands: yet, this was impossible as, for over half of the sample, the man was deceased. Research participant number seven epitomized the typical widowed respondent when she answered “(…I am) not really interested in sex unless (name of the deceased husband) came back” to the question “Would you like to be (sexually active)?” Overall, while our research participants appeared to be generally healthy from a physical standpoint and most of them had sexual desires, their desire to actually engage in interactional sex was mainly linked to the availability of a suitable partner, who was typically thought of as a husband. Four out of 13 women reported being interested in engaging in sex; one woman stated that she would consider engaging in it; however, nobody was available. Participant number 6 had interesting responses related to all the themes considered together. When asked about her feelings about changes in her sexuality as a product of getting older, she responded “Yes, I feel good as I get older. My husband really loved me.” Concerning her sexual desire, she answered “Sometimes, I just think of him (my husband). No one could ever change me (regarding sexuality changes)” and, as to whether she would like to be sexually active, she stated “No. Not really. Who(m) would I love?”

## 4. DISCUSSION

In the present study, we attempted to get a glimpse into the sexuality of older African American women. The shortness of the answers received to our questions was particularly striking; nonetheless, we gathered interesting (albeit very succinct) information and were able to identify four major themes that emerged from the coding procedures. Regarding the most endorsed theme, *having sexual desire (often unfulfilled)*, eight women out of 13 reported having sexual desire (those without a partner did not do anything about fulfilling this desire), four had none, and one reported missing her husband but not having any particular desire to engage in sexual interaction. This highlights the importance of the assertion made by Hooks [32] who strongly stated the necessity for African American women to challenge the perceptions of sexuality as a chore or a duty rather than a self-reinforcing pleasurable activity by creating a space in which to openly acknowledge and identify their own sense of sexuality. Perhaps (although not verifiable herein), the often traumatic introduction to intercourse experienced by their ancestors during decades of violent rape and abuse in the African American slavery era set the tone for what seems to be a denial of sexual desire and pleasure in some of our research participants. This may be particularly prevalent for those women born during the 1930s and 1940s, a time of racial and gender oppression, exacerbated by hearing stories from their mothers and grandmothers about the severity and cruelty of the slave trade.

Moreover, the public image of African American women’s role in the national labor force changed as the economy’s needs changed. In the mid-1970s, the image of the emasculating African American woman was portrayed as the root of the “pathology” of African Americans [33–34]. As a result, African American women’s sexuality was more and more regulated. With the transition from the industrial to the electronic age in the era of global capitalism, we have witnessed the dissolution of the social contract and the promulgation of the stereotype of African American women as “welfare queens” and carriers of “crack babies” (Williams [35] p.75). No longer associated with the domestic labor pool in the U.S., older African American women typically are no longer considered desirable. This shift in the economy leads to the common dismissal of their sexuality and could be internalized by some older African American women, who may dismiss their sexuality and desire altogether. Yet, this was not the case for the majority of our sample.

Regarding theme 2, *engaging in less sexual activity in older age*, lower levels of older African American women’s sexual activity in later life could be due, at least in part, to several cultural and socio-political issues. For example, according to Douglas [36], African American people’s perception of sexuality was constructed on a Whiteness model resulting in the silencing of the informed verbalization process that is needed to confront the taboo subject of African American sexuality (especially in older age). Collins [37] contended that African American women’s sexuality was defined by the system of enslavement in which sexual activity was controlled to enhance and supervise fertility. This set the stage for viewing older African Americans as sexless, as they were no longer useful for fertility purposes. A centuries-long tradition of dictating African American women’s sexuality exists in the U.S. through state-sanctioned reproductive exploitation for the purposes of labor; restrictive eugenics policies that enforce court-mandated sterilization of African American women; control of the image of these women through media and other social rhetoric; as well as the coercion of African American complicity through terror tactics historically employed by Whites against African Americans in the U.S. [38–40]. The commodification of African American sexuality through oppression and exploitation can naturally lead to a perception of older African American women’s sexuality as undesirable, and these women themselves may hold such perceptions.

Besides menopause and its repercussions often affecting sexual activity and desire adversely [41], negative age-related changes in levels of sexual activity in our sample may also reflect our respondents’ possible repulsion toward sex in older age, after having been in an intimate, exclusive relationship with a long-term sexual partner when they were relatively younger (however, our questions did not cover this issue). Another reason for not engaging or being interested in interactional sexual activities in older age could very well be a fear of sexually transmitted diseases (STDs). The risk of STDs is a threat to the health of African American women, as they typically report proportionately higher prevalence of concerns regarding the Human Immunodeficiency Virus (HIV) and STDs than any other racial/ethnic groups [42]. There is common belief that HIV is not a problem in older age but, with older populations living longer, increasing numbers are living with HIV or engaging in risky behavior. Indeed, the number of new HIV diagnoses is dropping among those 30 and younger, but is increasing among individuals who are 60 and older. The proportion of older people with HIV from heterosexual sex is slightly higher than that of the general population. One in 10 Americans living with the acquired immunodeficiency syndrome (AIDS) is over age 50, and it has been estimated that many people living with HIV will be over age 50 in the near future [43]. In prospective studies, investigators should inquire about the level of effort made by African American older women for protection from contracting the aforementioned diseases and how this issue impacts their sexuality.

A main reason for the findings relative to the aforementioned theme 2 was lack of available sexual partner, which is the focus of theme 3, *experiencing changes in one’s sexual life as a function of absence of a spouse*. One reason for its emergence is likely to be the difficulty experienced by older African American women (often reported by older women from other ethnic backgrounds) if attempting to find a suitable intimate partner. Carr [44] noted the “demographic obstacle” for older women to remarry (and to date) due to women greatly outnumbering men as they age. According to Carr, bereaved older women may not feel the need to pursue romantic relationships after the loss of their spouse because they may experience a sense of independence, freedom from caregiving, as well as sufficient financial stability due to Social Security, pension benefits, or other means of support. In an AARP study, several individuals aged 65 and older declared a desire to engage in interactional sex, but their major complaint was that their intimate partners were not interested. In particular, many of the men were not sexually active due to health and medication complications, and were resigned to the fact that they could not have sex anymore [22]. Although they verbalized having sexual desire, five of our research participants felt unsatisfied due to lack of an available intimate partner. Some respondents no longer had sexual desire; one woman reported that she had had no sexual desire since the death of her husband. In her own way, she had managed to cut off all her sexual feelings and was not aware of having any sexual desire. A few of the respondents stated that they missed their husbands, longing for their lost intimacy at all levels, beyond the sexual/physical aspect of the relationship. Some women made it clear that they had no intention of acknowledging any sexual desire now that their husbands were dead, corroborating our findings on denial of sexual desire in theme 1.

Some respondents reported that they kept sex away from their focus by engaging in prayer or seeing friends when feeling sexually deprived. We could speculate that the women who had no sexual desire since their husband’s death employed denial when sexual desire arose. However, a number of participants stated that they did not do anything to cope with feeling sexually deprived. Implementing coping strategies to fend off unfulfilled sexual desire could provide help to older women of all ethnic backgrounds when sexual interaction is not an option. Some of the women specifically longed to be in an intimate relationship, but finding a companion in older age might not be easy. Unfortunately, as previously mentioned, for unattached older women who long for an intimate companion, the opportunities to find suitable partners in later years become more and more dismal. Indeed, Jacoby [45] reported that “finding a partner” heads the list of what women 75-plus say would improve their sex life, but opportunities are limited for heterosexual older women who are interested in being sexually active. According to a 2005 study conducted by the Department of Health and Human Services [46], two thirds of physicians’ women patients were sexually active, but older women outnumber older men and, with each year of advanced age, the heterosexual partner gap widens. Concerning our target population in particular, older African American women have a greater shortage of men than all other ethnic/racial groups, largely due to higher mortality rates for African American men than African American women. In 2000, African American males had an average life expectancy at age 65 of an additional 14.5 years, yet African American women at age 65 had a life expectancy of 17.4 additional years. Moreover, in 2004, older men were much more likely to be married than older women (72% of men versus 42% of women). With higher life expectancy, high proportions of women are widows and live alone, as reported by the U.S. Department of Health and Human Services [47]. At 75 and older, when more than four out of five women are widowed, the percentage of women who had gone six months without intercourse was virtually identical to the percentage of widows. According to Jacoby [45], widows who do not wish to remarry yet believe that any type of sex outside marriage is morally wrong face particularly strong barriers to any sexual relationship. Although not verifiable herein, this could have been reflected in our findings on this theme.

Regarding theme 4, *exercising control over how one’s sexual life is conducted*, from a historical viewpoint, this is likely to be a sensitive topic for older African American women. The institution of slavery took place in an era when sexuality was most often a source of shame and even danger. In these times, the sexuality of African American people was typically labeled with emotionally-laden, derogatory terms, depicting African American sexuality as something exotic or forbidden, thus causing a rift to be formed between African Americans and Whites [36]. According to Hooks [48], under the breeding system, acts of cruelty involving both physical and psychological abuse were perpetrated on barren African American women. Furthermore, relief from the pressure to reproduce came only at an advanced age, when one was no longer exploitable for profit due to infertility. Older African American enslaved women lost “value” as they matured past their sexual, reproductive, and laboring prime. Indeed, with aging comes the freedom to do more of what one pleases. This includes simplifying and decluttering one’s life, letting go of possessions and excessive obligations, as well as feeling free to express thoughts and to be more in control over one’s destiny and body in particular. Some of our research participants volunteered that they felt more autonomous at this later stage of life and, thus, freer from pressures to engage in sexual activity unless they wanted to. Merging all themes with her answers, a widowed respondent shared that she enjoyed how her sexuality changed with age. She had tender words towards her deceased husband, thought of him when having sexual desire and remembered how much he loved her during those moments. She also made it clear that she would only love him. This woman stated that “No one could ever change me” when asked about potential changes in sexuality in older age. This is indicative of feeling in control over her aging sexuality and over having to deal with cessation of sexual interaction due to spousal loss.

In sum, some of our thematic results corroborate the findings of other researchers (e.g., lack of interest in interactional sex by older women residing in 29 countries [49] and the fact that, often, there is “no partner” with whom to have sex among primarily Caucasian older women [50]), extending these prior findings to older African American women. Our results on sexual desire (with over two thirds of our sample reporting having often-unfulfilled sexual desire) conflict with prior empirical evidence on other ethnic/racial populations (e.g., the low sexual desire findings of Smith and colleagues [50]). Exercising control over one’s sex life, an issue that was raised by a few of our respondents (without being solicited), is an understudied theme that should be the focus of more in-depth investigation.

### 4.1 Limitations and the Need for Future Research

Although the present findings are interesting, our investigation has many limitations. In this study, we combined quantitative and qualitative research elements in an attempt to gain preliminary insights into the sexuality of this neglected research population, so the fact that the sample size was limited is an acceptable circumstance at this stage of the research process and given the methodology implemented. However, the almost exclusive heterosexual composition of the sample was a limitation, in addition to the lack of quantitative data (other than demographic and health status information), and a potential selection bias, which was due to the fact that we had to locate research participants who agreed to engage in a discussion of sexual topics. Due to the very intimate nature of the study, our respondents were likely to be more open-minded about sexual issues than traditional older African American women (we also did not keep track of the number of women who declined to participate in this research). Furthermore, because the study’s design is cross-sectional, its findings do not imply causation. Also, many variables that are likely to be related to sexuality were not assessed, including (but not limited to) psychological health, body image, and use of medications with sexual side effects. Furthermore, we did not assess sexuality comprehensively. As already mentioned, we attempted to conduct this research in a manner that was as tactful as possible, to put our respondents at ease and reduce attrition that could have stemmed from asking them more detailed sexual questions (on topics such as masturbation or specific sex acts).

Most notably, despite our efforts to make research participants feel comfortable, many of the answers provided to our questions were very brief and did not allow for an in-depth exploration of the sample’s sexual attitudes and practices. As discussed more in depth in the following section, this might have been due to factors including embarrassment and discomfort associated with discussing sexuality in older age with a stranger and/or race-specific issues such as the harmful effects of gender oppression perpetrated against African American women of all ages. Although not verifiable in the present study, perhaps our participants engaged in Hines’s [15, 51] aforementioned notion of a “culture of dissemblance” as a coping strategy, which involves behavioral and attitudinal stances that provide a façade of disclosure and openness but, in reality, hide the truth of women’s inner lives in an attempt to reduce dominant negative stereotypes.

Another limitation of our investigation is that the data collected did not provide information regarding whether and how older African American women are reacting to current racialized stereotypes of sexual deviancy. The same can be said for the other side of this coin, i.e., the “politics of respectability,” which is allegedly aimed at shielding African American women from the negativity of those stereotypes [52]. However, these politics are used by upper class African American women (and men) to control the behaviors of African American women who dare challenge normative sexual desires by rejecting the acceptable, sanitized versions of women’s sexuality [53–54]. These controversial, yet important, issues could be further researched by scholars interested in clarifying how much of the sexuality of older African American women is being suppressed in order for them to appear respectable, who is controlling this process, and what means are being used to achieve this, as well as whether these women are aware of this situation and resent such oppression.

Given the uniracial focus of this preliminary study and the limited sexual information gathered via our interview protocol, we are not in the position to assess whether older African American women’s tendency to dismiss their sexual desire is a phenomenon applicable to the same extent to other races. More studies are certainly needed on this neglected research topic, in order to clarify and strengthen the concept of older African American women’s sexual politics as ethnically distinct. Similarly, given the very little information gathered on the small lesbian/bisexual component of our study, we are unable to make distinct comments on older African American lesbians or on older African American bisexual women. Future investigations should be aimed at these research populations, which certainly need more attention and visibility in research studies.

### 4.2 Challenges and Potential Solutions in Regard to Conducting Sexuality Research on Older African American Women

The paucity of sexuality data gathered in the present investigation raises several methodological questions, such as: are the minimal narratives that we were able to draw from respondents a product of the challenges of collecting data on the topic of sexuality in older age? And, if so, what were the challenges? Indeed, as already mentioned, an exploration of sexuality topics in older age could be difficult, for instance because the research population in question may not be comfortable with the subject matter, possibly feeling “exposed” when discussing sex issues. What could be the best approach to a topic that is so profoundly intimate, and how might researchers overcome the aforementioned obstacles? In the present section, we have succinctly discussed these topics and have attempted to provide potential solutions so that interested researchers will be equipped to avoid running into the same challenges.

First, it must be kept in mind that, in general, many ethnic minority groups do not trust researchers. In this regard, Haynes and Hatch [55] posited that the obstacles faced in obtaining minority participants during the Women’s Health Initiative (WHI), a multi-centered clinical trial for women aged 50 – 79 to determine the efficacy of low-fat diets, hormonal replacement therapy, and vitamin D/calcium supplementation to prevent several diseases, highlighted the long-standing problems associated with recruitment of older minority women in clinical trials. It was noted that “the Tuskegee” effect, i.e., a United States Public Health Service (USPHS) syphilis study at Tuskegee involving abuse of minorities, has often been cited as a reason for the non-participation of African Americans in health studies. Recently, Katz [56] stated that the fallout from the USPHS Syphilis Study at Tuskegee included a longstanding national belief that African Americans would refuse to become research study participants. This belief was given credence by a 7 – 10 minute short survey of WHI clinic respondents: results showed that, compared to 4% of White women, about one-third of African American women were distrustful of scientists.

Regarding strategies to enhance research recruitment of African Americans regardless of age and research topics, higher research participation has been noted when African American women were recruited from public housing projects, churches, and the National Black Women’s Health Project, all central places that are mainstays in the African American community [55]. According to the aforementioned authors, studies such as the National Cancer Institute’s Breast Cancer Prevention Trial highlight the important role that physician and family support play in research studies’ recruitment and participation. Also, the concept of having multiple partners sponsor a proposed research project could yield success. The authors offered an example from the Office of Research on Women’s Health, that of “Heart, Body and Soul,” a project providing prevention services and health education in east Baltimore, Maryland, where a collaborative partnership between Johns Hopkins University and local religious leaders fostered acceptance of the program. Without having these strategies in place, it is hard for researchers to create a situation in which African American women would feel comfortable discussing intimate sexual issues. Also, maybe the measures and the procedures that we used (such as a short, structured interview protocol administered with a few other assessment tools usually in a public place, with limited time available) might have been a deterrent to sexual disclosure in our study. As pointed out by Rose in 2003 [57], African American women of all ages typically look for trusted friends within their community, seeking advice and a safe space in which to share intimate concerns without having to consider someone else’s agenda, expectations, or needs. On the other hand, our research assistants interacted with our respondents with a research agenda, having to collect data for a university-sponsored project that was not previously known to the participants, nor sponsored by an African American organization or an African American health provider.

Rose’s sexuality research work [57] could be used as an ideal template to utilize in future sexuality studies on our target population. In research on the sexuality of African American women, Rose approached her interviewees as an open process centering on their sexual experiences and reflections, as well as their perceptions of societal issues relating to race and gender. She made sure not to use a fixed list of questions, choosing instead a conversational style, and asked research participants the reasons why they agreed to participate in sexuality-focused research. This last question, although ideal, is not easily applicable to small sex studies conducted by RAs such as our investigation, in which finding willing participants was a hard task. In the case of our RAs, asking willing respondents to articulate their reasons for wanting to participate in the study could have provoked the undesirable response of withdrawing from the research project, thus it was not mentioned. Moreover, our interview protocol did not have a developmental structure. On the other hand, in the case of Rose’s study, once all the aforementioned topics were covered, she proceeded to inquire about issues such as intimacy, how respondents learned about sexuality, masturbation, orgasms, as well as their experience of first menstruation, virginity, pregnancy, and motherhood. Rose also investigated sexual abuse and race in relation to topics including sex, sexism, sexual fantasy, and respondents’ regret, while we did not do so. Importantly, the shape of each of Rose’s questions emerged from these conversations, not being dictated by the research team. Interestingly, her respondents often offered stories covering the aforementioned topics well before she mentioned them.

Rose’s [57] in-depth way of conducting sex research on African American women is certainly cumbersome, but if researchers take on the challenge, their research findings could be rich and comprehensive. When comparing her research approach to ours, in addition to the series of limitations already discussed, we faced additional constraints that precluded us from adopting a comprehensive model of interviewing. Most notably, the structured interview protocol followed by our RAs did not include, at the end of each question, a note for the interviewers to make statements such as “tell me more,” or anything that reminded them to prompt respondents to reveal more information. Moreover, the interviews did not last long, often being only ½ hour long or even shorter, depending on how much the respondent chose to disclose. On the other hand, Rose’s conversations lasted about two to three hours and took place in private, while our interviews were often conducted in public places such as libraries. Moreover, at Rose’s request, several of her respondents made up pseudonyms and were able to find themselves in print, thus likely feeling a strong connection to the published version of their sexuality stories. Therefore, another strategy to be implemented in future research is to offer the opportunity for African American older women to eventually locate their stories in published manuscripts. This could be very rewarding, as respondents would likely feel honored and proud of their research participation. They could show to their loved ones that they (using a safe pseudonym) contributed to important research. In the case of our study, we did provide a certificate of completion of the research to each respondent, but did not have in place a mechanism for creating pseudonyms, nor did we tell participants that they could have seen their revelations in press (of course, with the latter being carefully disguised to protect their anonymity).

When comparing our study to Rose’s [57] investigation, an issue that should be kept in mind is that older African American women’s upbringing was likely more conservative than that of younger generations, such as the interviewees in Rose’s study. Indeed, cohort differences could make it even harder to engage our target population in sexuality-focused disclosure. Concerning methodological challenges related to sexuality research on *older* women in particular, themes of silence and invisibility have tended to dominate sexuality in later life [58]. We as researchers must engage in debates around methodological issues of researching sexuality in older age. For example, in a qualitative study of women over the age of 70, Jones [59] found that many participants were concerned about the potential for misunderstanding and miscommunication regarding sex and sexuality. Successful research approaches to address this issue included to establish a shared meaning at the beginning of the research encounter between researchers and participants and to use common colloquial language familiar to the respondents. Therefore, adopting Jones’s approach could help avoid language confusion around sexuality. Also, according to Jones, individual and telephone interviewing were preferred over group interviews due to the perceived sensitive nature of the questions asked during the interviews. Some respondents favored the idea of focus groups as a way to empower participants to discuss sexuality and aging through support and encouragement. Moreover, participants felt that knowledge of the questions prior to data collection would be helpful and empowering. In future studies, researchers should consider whether individual versus group or telephone interviews, as well as focus groups, would be the best way to collect data for their sexuality-focused research projects. Perhaps, when conducting research involving sensitive topics targeting older African American women (as well as older women of other ethnic backgrounds), survey research in which anonymity of the respondents is maintained or assured could significantly improve the response rate and content. Respondents might be more open to expressing their opinion regarding these sensitive topics when completing surveys than in face-to-face or even in telephone interviews.

Another one of our potential methodological challenges was that maybe older African American women prefer disclosing sexual topics to professionals who are not young RAs. The desire to have more mature professionals who specialize in the gerontology and the health field conduct the interviews (rather than students) may have held back the participants in our study from providing intimate details on their sexuality. In this regard, Hahn [60] discussed full disclosure from the participants of a 2009 study of 85-year-old women who lived alone in their own residences. In addition to the principal investigator, the interview team included a community health nurse with 30+ years of gerontological experience, as well as a research nurse. Thus, their research participants may have revealed details about their sexuality based on the belief that the study was conducted by experts in the field of sexuality research. On the other end, in our study, the RAs were typically in their early 20s and had a limited amount of training in sexuality. Moreover, ethnicity is a known potential confounding variable in the interview process, with respondents giving socially desirable answers to interviewers of other races, *except* when the interviewers occupy a higher status role [61]. Could educational status have played a similar role in our study, if research participants had the perception that the interviewees were of a different socio-economic status and thus felt intimidated? This latter point conflicts with the prior discussion on expertise and maturity, but it is nonetheless a factor to consider. The aforementioned considerations regarding age, expertise level, and socio-economic status of the RAs/interviewers in relation to African American older women’s disclosure of sexuality issues should be kept in mind when conducting sex research with our target population. These issues, in addition to the methodological concerns discussed earlier in this section, might have played a significant role in our study. In sum, based on our experience, employing young RAs who a) have an often-higher financial status than the older African American women interviewed, b) are using a short, structured interview protocol without follow-up options for each question, c) have limited time to complete the sexuality-focused interview, and d) administer the research assessment materials (including the interview) typically in public places such as libraries, is not the best way to induce sexual disclosure in African American older women.

## 5. CONCLUSION

As stated by Hooks [48], the first sexual roles of African American women’s bodies were commodities, units of labor, as planters besieged by “virulent attacks on slave importation” turned to breeding slaves to encourage profit. Moreover, enslaved women were painted as overtly sexual and amoral animals, with natural inclinations for domestic and field labor, which reified the abusive system. It is possible that this history contributed to why the older African American women in the present study were very reserved about sharing information concerning their sexuality. Likely due also to some of the methodological challenges discussed above, our respondents provided brief, yet revealing answers to sexuality questions in one-on-one interviews with RAs of the same gender and race (yet much younger in age than the respondents). Four major themes that emerged from the content analysis were identified and discussed; nobody reported having any sexual problem. Nevertheless, would older African American women be likely to seek professional help if they perceived that their sexual functioning was impaired? To answer this question, we must consider that, although some older adults continue to be sexually active well into later life, health care professionals seldom address sexual functioning with older patients who, in turn, rarely raise sexual concerns with a health care provider. In this regard, an AARP study revealed that only 28% of men and 14% of women 45 years of age and older had ever sought treatment from personal physicians or other health specialists for any problems related to sexual function [22]. People 45 and older report that books — not health professionals — are their primary source of sex information and, as already noted, older women are particularly reluctant to seek treatment for physical conditions that are impacting their sexuality adversely [45]. Although this topic was beyond the scope of our study, judging from the brevity and content of our respondents’ answers, perhaps, even *if* an older African American woman had recognized the need for professional attention to a sex problem, she might have not been comfortable discussing how to address this problem with her health providers. The cause could be any of the above-mentioned factors, including a sense of privacy especially valued within the African American community, wherein secrecy has historically been a necessary requirement for survival.

In conclusion, based on the lessons learned from conducting our sexuality study, we have attempted to provide the reader with a variety of useful recommendations on how to avoid receiving short sexuality-related answers; for instance, Rose’s [57] insightful and creative ways of investigating sexuality among African American women, in general, could guide future studies on older African American women in particular. Paying attention to respondents’ initial experiences of sexuality, including how it took shape and evolved with age, the complexity of their race, gender, class, and sexuality should be explored simultaneously in future studies, to better elucidate the themes identified in this discussion. Often viewed in manufactured images of the “jezebel and sexual savage,” African American women have responded in many ways to the aforementioned potentially traumatizing circumstances, including keeping discussions of and attitudes toward sexuality to a minimum, thus becoming more conservative and sexually restrained. Social variables such as a widespread propensity to equate aging with senility and poor health, as well as an age-related decline in both beauty and a positive sense of self, could further complicate this picture. In 2006, Slevin [62] contended that older age is viewed as “a social contagion” that could compel older adults to avoid one another and seek the company of those younger than themselves. These ageist attitudes are likely to apply to older African American women as well. In future research, it would be very interesting to identify the creative ways through which some older African American women are able to counter the multiple negative pressures that are typically inscribed on their sexuality, and how they manage to nonetheless feel empowered in their sexual expression. Perhaps, although still unbeknown to researchers such as ourselves, many older African American women have found innovative ways to integrate their sexual desire with sexual activity within their lives, as sexual pleasure is a main component of integral sexual health [63]. These women would provide powerful role models for future generations of African American women.
